# Energy Stress-Mediated Cytotoxicity in Tuberous Sclerosis Complex 2-Deficient Cells with Nelfinavir and Mefloquine Treatment

**DOI:** 10.3390/cancers10100375

**Published:** 2018-10-10

**Authors:** Henry D. McCann, Charlotte E. Johnson, Rachel J. Errington, D. Mark Davies, Elaine A. Dunlop, Andrew R. Tee

**Affiliations:** 1 Division of Cancer and Genetics, Cardiff University, Heath Park, Cardiff CF14 4XN, UK; McCannHD@cardiff.ac.uk (H.D.M.); johnson.ce2010@gmail.com (C.E.J.); ErringtonRJ@cardiff.ac.uk (R.J.E.); DaviesDM@cardiff.ac.uk (D.M.D.); DunlopEA@cardiff.ac.uk (E.A.D.); 2 Department of Oncology, South West Wales Cancer Centre, Singleton Hospital, Swansea SA2 8QA, UK

**Keywords:** mTOR, nelfinavir, mefloquine, TSC, cancer therapy, energy homeostasis, ER stress

## Abstract

To find new anti-cancer drug therapies, we wanted to exploit homeostatic vulnerabilities within Tuberous Sclerosis Complex 2 (TSC2)-deficient cells with mechanistic target of rapamycin complex 1 (mTORC1) hyperactivity. We show that nelfinavir and mefloquine synergize to selectively evoke a cytotoxic response in TSC2-deficient cell lines with mTORC1 hyperactivity. We optimize the concentrations of nelfinavir and mefloquine to a clinically viable range that kill cells that lack TSC2, while wild-type cells tolerate treatment. This new clinically viable drug combination causes a significant level of cell death in TSC2-deficient tumor spheroids. Furthermore, no cell recovery was apparent after drug withdrawal, revealing potent cytotoxicity. Transcriptional profiling by RNA sequencing of drug treated TSC2-deficient cells compared to wild-type cells suggested the cytotoxic mechanism of action, involving initial ER stress and an imbalance in energy homeostatic pathways. Further characterization revealed that supplementation with methyl pyruvate alleviated energy stress and reduced the cytotoxic effect, implicating energy deprivation as the trigger of cell death. This work underpins a critical vulnerability with cancer cells with aberrant signaling through the TSC2-mTORC1 pathway that lack flexibility in homeostatic pathways, which could be exploited with combined nelfinavir and mefloquine treatment.

## 1. Introduction

Mechanistic (formally mammalian) target of rapamycin (mTOR) is often referred to as a master regulator of cell growth [1]. mTOR is a serine/threonine protein kinase that coordinates cell growth, metabolism, protein synthesis and autophagy as a protein kinase complex, termed mTOR complex 1 (mTORC1). mTORC1 is fundamentally involved in the build-up of cellular biomass, which is rate-limiting for hyper-proliferative cancer cells. As well as cell growth control, mTORC1 promotes metabolic transformation, neovascularization, cell survival and metastasis (reviewed in reference [2]). Genetic mutations leading to mTORC1 hyperactivation often occur in sporadic cancer but also underlie several tumor predisposition syndromes, such as Tuberous Sclerosis Complex (TSC) [3] and Cowden disease/PTEN hamartoma syndrome (reviewed in reference [4]). mTORC1 hyperactivity has also been identified in numerous cancer types including lung (>50% in both small cell and non-small cell lung cancer cases [5,6]), ovarian (approximately 70% of ovarian cancers [7]), renal cell carcinoma [8] and head and neck cancers [9]. Although uncommon, activating mutations within the kinase domain of mTOR occurs in cancer [10,11]. More frequently, genes that function upstream of the mTORC1 signaling pathway are mutated in cancer, such as PI3K, PTEN and RAS, which then cause aberrant signal transduction through mTORC1 to drive cancer growth [12].

TSC is an autosomal dominant condition caused by mutations in either TSC1 or TSC2 and is characterized by mTORC1 hyperactivity, tumor growth in multiple organs, neurocognitive problems and epilepsy [13]. TSC1 and TSC2 form a tumor suppressor protein complex that functions as a GTPase-activating protein (GAP) towards the small G-protein, Ras homologue enriched in brain (Rheb) [14,15]. When associated with TSC1, the GAP function of TSC2 switches Rheb from its active GTP-bound state to an inactive GDP-bound state to turn off mTORC1. Consequently, loss-of-function mutations in either TSC1 or TSC2 cause Rheb to become predominantly GTP-loaded, thereby constitutively turning on mTORC1. mTORC1 inhibitors, such as rapamycin analogues (rapalogues), are currently used as the first-line therapy to treat TSC and are effective at shrinking tumors and delaying disease progression. However, mTORC1 inhibitors are cytostatic meaning that TSC tumor cells regrow after treatment withdrawal (reviewed in reference [16,17]). This cytostatic nature of mTOR inhibitors is a principal reason why rapalogues have had limited clinical success in the cancer setting, where the lack of cytotoxicity leads to acquired drug resistance (reviewed in reference [2]).

To overcome the shortcomings of directly targeting mTORC1 in cancer, an alternative therapy might be to exploit the intrinsic vulnerabilities of cancer cells that have constitutive mTORC1 activation. In principle, you could take advantage of the cancer’s inability to restore homeostatic balance during prolonged periods of drug induced cell stress to trigger a cytotoxic response. In contrast to cancer cells, non-cancerous cells would have greater flexibility in their homeostatic pathways, allowing them to better tolerate drug treatment. As loss of TSC1/2 and mTORC1 hyperactivity results in an increased burden of unfolded protein within the endoplasmic reticulum (ER), which causes membrane expansion of the ER and activation of the unfolded protein response (UPR) [18], a possible therapy would be to employ ER stress inducers. To enhance the cytotoxic effect, you could also target cell survival pathways, such as autophagy. Previously, we examined the combined effect of chloroquine (an autophagy inhibitor) with nelfinavir (an ER stress inducer), which selectively enhanced cell death in mTORC1 hyperactive cells [19]. Nelfinavir is a clinically approved HIV inhibitor that induces ER stress and shows promise as an anti-cancer agent [20]. While these clinically viable drugs were previously shown to be selectively cytotoxic to mTORC1-active cells, combined chloroquine and nelfinavir treatment was not sufficient to kill all the cells, showing a heterogeneous drug effect and a significant level of drug resistance. In this current study, we wanted to refine our previous combinatory drug therapy to enhance their cytotoxic potency. To do this, we considered dual treatment with nelfinavir and mefloquine, which appeared to kill a higher percentage of *Tsc2*-deficient cells when compared to nelfinavir and chloroquine in our initial analysis [19]. Mefloquine is a chloroquine derivative used in both the prevention and treatment of malaria and unlike chloroquine, more readily crosses the blood-brain barrier. Mefloquine has been reported to inhibit autophagy (via blocking the formation of autophagosomes [21]), to induce reactive oxygen species (ROS) [22] and to prevent PI3K/Akt/mTORC1 signaling [23]. In this study, we demonstrate that nelfinavir and mefloquine show therapeutic promise as a drug combination that has high potency to kill TSC2-deficient cells and cancer cells with high mTORC1 activity.

## 2. Results

### 2.1. Nelfinavir and Mefloquine Synergize, and Selectively Kill Tsc2-Deficient Cell Lines

**Figure 1:**
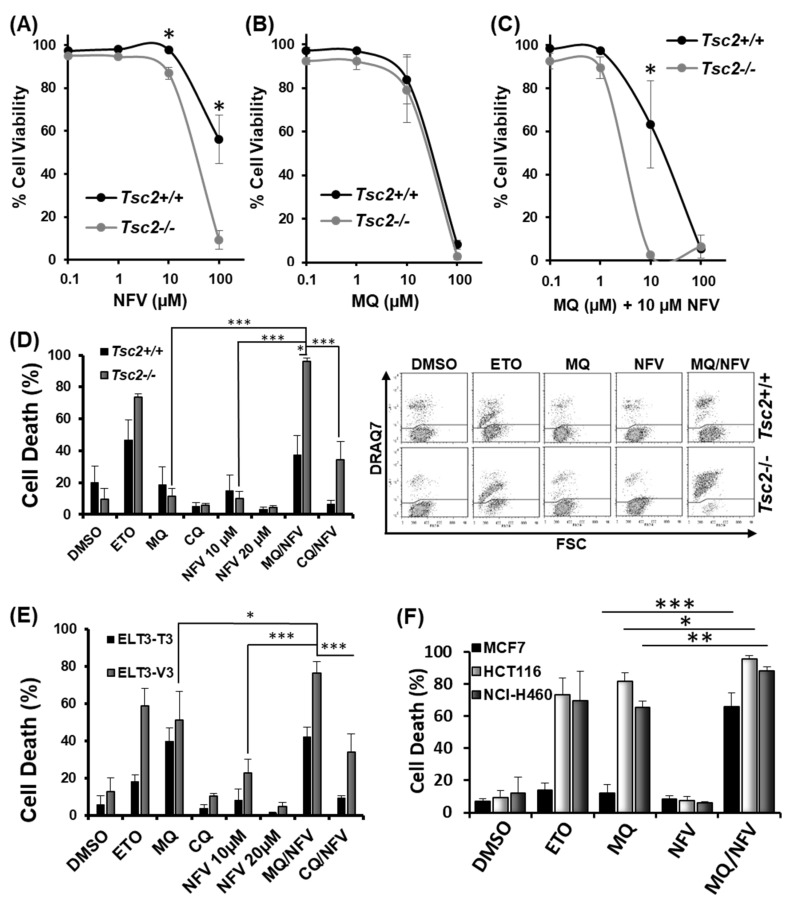
Mefloquine and nelfinavir synergize to kill *Tsc2*−/− Mouse embryonic fibroblasts (MEFs), ELT3-T3 and sporadic cancer cells. Dose response curves were performed in *Tsc2*+/+ and *Tsc2*−/− MEFs using flow cytometry to measure cell death following treatment with (**A**) nelfinavir (NFV); (**B**) mefloquine (MQ) and (**C**) combined mefloquine with a fixed concentration of 10 µM nelfinavir (MQ/NFV); (**D**) *Tsc2*+/+ and *Tsc2*−/− MEFs; (**E**) ELT3-T3 and ELT3-V3; (**F**) MCF7, HCT116 and NCI-H460 were treated with either DMSO, etoposide (ETO), 10 µM mefloquine (MQ), 10 µM nelfinavir (NFV) or mefloquine combined with nelfinavir (MQ/NFV) for 48 h. Cells were then tested by flow cytometry and cells were separated into viable and non-viable cell populations via DRAQ7 staining. Statistical significance is shown with combination treated *Tsc2*−/− MEFs or the ELT3-V3 cells to their wild-type controls, and comparing single drug treatment of mefloquine and combination with the MCF7, HCT116 and NCI-H460.

To build on our previous study [19] and to further explore the impact of nelfinavir and mefloquine dual therapy, we tested the ability of nelfinavir and mefloquine to synergize and induce cell death. Loss of cell viability was quantified with escalating concentrations of drug, either as single agents or in combination using flow cytometry with DRAQ7 staining. DRAQ7 can only penetrate membranes of cells that are compromised (i.e., are no longer viable). Loss of cell viability (determined by an increase of DRAQ7 fluorescence) with single drug treatments for nelfinavir and mefloquine is shown in Figure 1A,B, respectively. To ascertain how synergistic this combination was, a range of mefloquine concentrations were tested with a fixed 10 µM concentration of nelfinavir (Figure 1C). Quantification of cell death was processed in CompuSyn using a non-constant ratio approach to generate a Combination Index (CI) value, where it is considered that a score <1 is synergistic, a score of 1 is additive, and a score >1 is antagonistic. Results reveal that 10 µM mefloquine was synergistic with 10 µM nelfinavir to induce cell death in the *Tsc2*−/− mouse embryonic fibroblasts (MEFs) (CI value = 0.03). Due to this finding, further assays were carried out using 10 µM mefloquine and 10 µM nelfinavir. We observed selective cytotoxicity with the combinatory drug treatment in cells lacking *Tsc2*, i.e., (37.6% −/+ 11.8 standard deviation (St-Dev) in the *Tsc2*+/+ MEFs versus 96% −/+ 2 in the *Tsc2*−/− MEFs) (Figure 1D). Etoposide was employed as a positive control and induced cell death in both the *Tsc2*+/+ and *Tsc2*−/− MEFs.

To confirm that the genetic loss of *Tsc2* was responsible for selective cell death, mefloquine and nelfinavir were then tested in ELT3 (Eker rat leiomyoma-derived) cells (Figure 1E), another model of TSC [24]. Results were comparable to that observed in the *Tsc2*−/− MEFs, where ELT3-V3 cells (lacking *Tsc2*) had a significant induction of cell death (76% −/+ 6.3) when compared to the rescued ELT3-T3 with *Tsc2* re-expressed (42% −/+ 5.4). To determine whether combined nelfinavir/mefloquine drug treatment could also target sporadic cancer cell lines, we tested three mTORC1-hyperactive cancer cells (Figure 1F). Results from flow cytometry showed that combined 10 µM nelfinavir with 10 µM mefloquine treatment caused a high degree of cytotoxicity in the MCF7 breast cancer cell line (67% −/+ 8.30), in the HCT116 colorectal cancer cell line (96% −/+ 1.95), and in the NCI-H460 lung cancer cell line (89% −/+ 2.5). As single drug treatments, nelfinavir and mefloquine were not cytotoxic to MCF7 cells. As a mono-agent, mefloquine was observed to potently kill the HCT116 and NCI-H460 cell lines (but to a lesser degree when compared to combination treatment with nelfinavir). Etoposide was used as a control, which was effective at killing the HCT116 and NCI-H460 cells, while the MCF-7 cells were notably resistant.

### 2.2. Combined Nelfinavir and Mefloquine Treatment Inhibits Tumor Formation, Induces Cytotoxicity in Tsc2−/− Spheroids, and Prevents Spheroid Outgrowth

**Figure 2:**
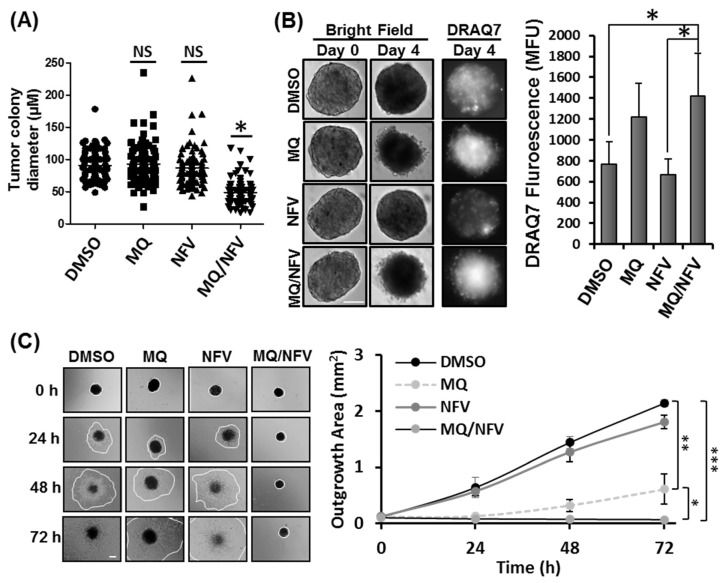
Mefloquine and nelfinavir prevents colony formation and spheroid growth. (**A**) Colony formation was tested in *Tsc2*−/− MEFs seeded on soft agar that were treated for 14 days with Dimethyl Sulfoxide (DMSO), 10 µM mefloquine (MQ), 10 µM nelfinavir (NFV) or in combination. Tumor diameters were measured using Image J; scale bar is 200 μm. Significance was observed when comparing combined nelfinavir and mefloquine treatment to DMSO vehicle control. (**B**) *Tsc2*−/− MEF spheroids were treated under the same conditions as (**A**) for 96 h. DRAQ7 was supplemented for the final 36 h to monitor cell death before images were taken and DRAQ7 fluorescence quantified. (**C**) Spheroids treated in (**B**) were re-plated onto standard tissue culture plates and grown in drug-free media. Images were taken every 24 h and the area of outgrowth calculated using Image J, scale bar is 200 μm and outgrowth area is graphed.

To determine whether nelfinavir and mefloquine combination treatment prevents the formation of tumors in vitro, *Tsc2*−/− MEFs were grown in soft agar in the continued presence or absence of drug over 14 days (Figure 2A). We observed that when combined, nelfinavir and mefloquine significantly reduced colony growth compared to single drug treatments, which as mono agents did not significantly reduce colony size. Average colony size was 49.1 µm +/− 19.27 in diameter after nelfinavir/mefloquine treatment compared to 90.7 µm +/− 22.12 observed in samples treated with dimethyl sulfoxide (DMSO). To examine whether drug treatments could induce cell death in established tumor spheroids, *Tsc2*−/− MEFs were grown as spheroids and once established were then incubated with either DMSO, nelfinavir or mefloquine (as single drug treatments or in combination) for 4 days (Figure 2B). To measure loss of cell viability, spheroids were DRAQ7-stained during drug incubation. Intensity of DRAQ7 fluorescence was higher after combined treatment with nelfinavir and mefloquine (1424 MFU (mean fluorescent units) −/+ 404), when compared to single drug treatment with nelfinavir (668 MFU −/+ 146), and DMSO (768 MFU −/+ 214). Elevated DRAQ7 fluorescence was also observed with single drug treatment with mefloquine (1216 MFU −/+ 328). Tumor spheroids were transferred to plastic tissue culture plates to assess recovery and outgrowth of cells after drug removal (Figure 2C). Cell outgrowth was measured over 72 h, and showed cell recovery from the tumor spheroid after single drug treatment with either nelfinavir or mefloquine, although cell outgrowth was much slower with mefloquine when compared to either DMSO or nelfinavir treatments. In contrast, cells were unable to recover after combined treatment with mefloquine and nelfinavir, as observed by no detectable outgrowth of cells over the 72 h period. These data reveal that the combination of mefloquine and nelfinavir is effective at killing the bulk tumor.

### 2.3. Homeostatic Balance is Lost in Tsc2−/− MEFs after Combination Treatment with Nelfinavir and Mefloquine

**Figure 3:**
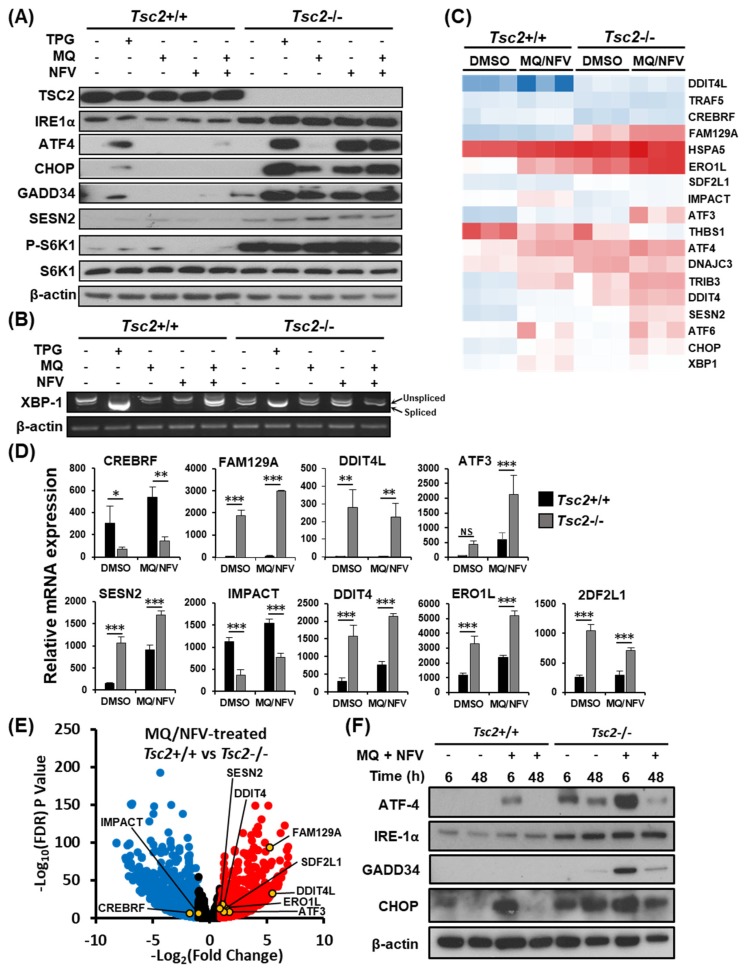
Mefloquine and nelfinavir drug combination causes increased ER stress in *Tsc2*−/− MEFs. (**A**) *Tsc2*+/+ and *Tsc2*−/− MEFs were treated with either DMSO, 1 µM thapsigargin (TPG), 10 µM mefloquine (MQ), 10 µM nelfinavir (NFV), or mefloquine and nelfinavir combination for 6 h, where indicated. Total protein levels of TSC2, IRE1α, ATF4, CHOP, GADD34, S6K1 and β-actin and S6K1 phosphorylated at Thr389 were detected by Western blot. (**B**) *Xbp1* mRNA splicing was determined from the same treatments as described in (**A**). PCR products were resolved on agarose gels (unspliced = 480 bp upper band, spliced = 454 bp lower band). (**C**–**E**) *Tsc2*+/+ and *Tsc2*−/− MEFs were treated with either DMSO or mefloquine and nelfinavir combination (MQ/NFV) for 6 h before being processed for RNA sequencing. A heat map for a panel of ER stress-linked genes is shown (**C**) and are graphed in (**D**). (**E**) Differences of mRNA expression between *Tsc2*+/+ and *Tsc2*−/− MEFs treated with mefloquine and nelfinavir is shown as a volcano plot and highlights ER stress genes. (**F**) *Tsc2*+/+ and *Tsc2*−/− MEFs were treated with DMSO or mefloquine (MQ) and nelfinavir (NFV) combination for 6 h and 48 h. Total protein levels of ATF4, IRE-1α, GADD34, CHOP and β-actin were determined by Western blot.

We postulated that the most likely mechanism of cell death would be because of prolonged ER stress and an inability to recover. Therefore, we initially examined ER stress signaling. Western blots were carried out on a panel of ER stress markers (inositol-requiring and ER-to-nucleus signaling protein 1α (IRE1α), activating transcription factor 4 (ATF4), C/EBP homologous protein (CHOP), growth arrest and DNA damage-inducible protein (GADD34), and Sestrin 2 (SESN2)), with thapsigargin treatment as a positive control. We observed a large difference in expression of ER stress markers when comparing *Tsc2*−/− to their wildtype *Tsc2*+/+ controls (Figure 3A). For instance, the *Tsc2*−/− MEFs have a higher level of IRE1α, ATF4, CHOP and GADD34 protein expression after thapsigargin and nelfinavir as single drug treatments, while mefloquine weakly induced ER stress. The control cells exhibit a lower basal level of ER stress and show little ER stress induction with single or combination treatments. Analysis of p70 ribosomal protein S6 kinase 1 (S6K1) phosphorylation showed that *Tsc2*−/− MEFs had a high level of mTORC1 activity that was not altered with drug treatments. To confirm that ER stress was enhanced after treatment, X-box binding protein 1 (*Xbp1*) mRNA splicing was determined (Figure 3B), revealing a higher level of *Xbp1* mRNA splicing in *Tsc2*−/− cells after combination drug treatment when compared to the untreated *Tsc2*−/− and treated *Tsc2*+/+ controls. We also observed a high level of *Xbp1* mRNA splicing in untreated *Tsc2*−/− MEFs, showing that these cells have a higher basal level of ER stress.

To assess transcriptional changes, RNA sequencing was carried out on mRNA samples generated from *Tsc2*+/+ and *Tsc2*−/− MEFs treated with either DMSO or nelfinavir and mefloquine. We observed elevated expression of ER stress genes in both *Tsc2*+/+ and *Tsc2*−/− MEF cell lines after combined nelfinavir and mefloquine drug treatment, which was higher in the absence of *Tsc2* (Figure 3C,D and Table S1). We also observed elevated expression of ER stress genes in untreated *Tsc2*−/− MEFs when compared to wildtype. Conversely, negative regulators of ER stress such as CREBRF and IMPACT were expressed at higher levels in the *Tsc2*+/+ MEFs when compared to the *Tsc2*−/− MEFs (Figure 3E and Table S2). The volcano plot illustrates the differential expression of genes involved in ER stress between the *Tsc2*+/+ and *Tsc2*−/− MEFs after combined nelfinavir and mefloquine treatment (Figure 3E).

To determine the capacity of *Tsc2*+/+ and *Tsc2*−/− MEFs to effectively restore ER stress during treatment, we examined the expression of ER stress proteins after combined nelfinavir and mefloquine treatment at an early 6 h time point and at a longer 48 h time point. The 6 h time point reflects acute ER stress induction after treatment, while the 48 h time point reflects ER stress recovery during prolonged drug treatment (Figure 3F). In wildtype *Tsc2*+/+ MEFs, nelfinavir and mefloquine treatment increased the expression of CHOP and ATF4 protein at 6 h. However, by 48 h the expression of ATF4 and CHOP was restored back down to a level equivalent to untreated cells, indicating a complete recovery to the ER stress signaling pathway in the *Tsc2*+/+ MEFs. Similarly, the *Tsc2*−/− MEFs also recovered from ER stress at the 48 h time point. The ability to restore the ER stress signaling pathway in the *Tsc2*−/− MEFs implies that the ER stress pathway is unlikely the main trigger that instigates the observed cell death response.

### 2.4. Cytotoxicity of Tsc2-Deficient Cells with Nelfinavir and Mefloquine is Energy Dependent

**Figure 4:**
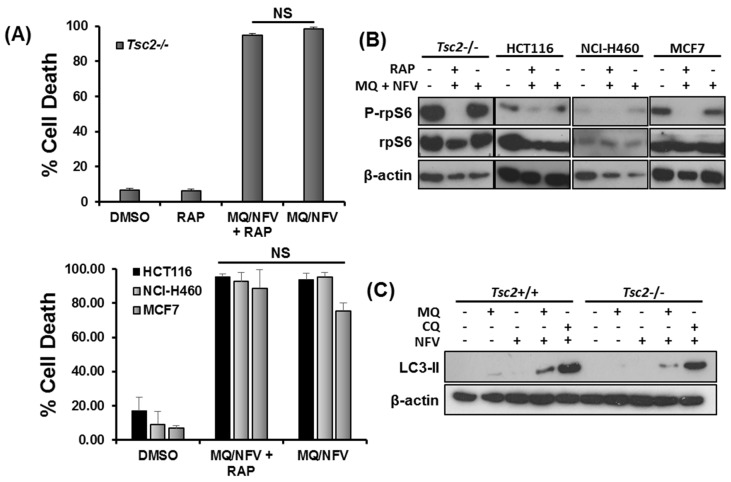
Mefloquine and nelfinavir drug cytotoxicity is not associated with mTORC1 hyperactivity and causes minimal autophagy inhibition. (**A**) *Tsc2*−/− MEFs, NCI-H460, MCF7 and HCT116 cells were pre-treated with 50 nM rapamycin (RAP) for 1 h, where indicated, before being treated with 10 μM nelfinavir (NFV) and 10 µM mefloquine (MQ) for 48 h. Cells were stained with DRAQ7 and % cell death determined using flow cytometry. (**B**) Western blotting was carried out to determine rp-S6 phosphorylation at Ser235/236 in the cells treated in (**A**) after 48 h of treatment. (**C**) *Tsc2*+/+ and *Tsc2*−/− cells were treated with DMSO, 10 µM mefloquine (MQ), 20 µM chloroquine (CQ), 10 µM mefloquine or 20 µM chloroquine combined with 10 µM nelfinavir for 3 h. Accumulation of lipidated LC3-II were analyzed by Western blot. Total protein levels of β-actin were used as a loading control.

We next wanted to determine whether mTORC1-hyperactivity was important for the induction of cell death. We carried out flow cytometry with DRAQ7 staining in *Tsc2*−/− MEFs, HCT116, NCI-H460 and MCF7 cells treated with either DMSO, or combination with nelfinavir and mefloquine in the presence or absence of rapamycin for 48 h (Figure 4A). Rapamycin failed to rescue cell death in these cell lines, showing that the cytotoxic effect of this drug combination is not prevented when mTORC1 is inhibited. Control western blots showing that rapamycin treatment was sufficient to block ribosomal protein S6 (rpS6) phosphorylation in these cells are shown (Figure 4B). As mefloquine is classically known as an autophagy inhibitor, we next analyzed build-up of lipidated LC3 to measure autophagy inhibition after treatment with either mefloquine or chloroquine after 3 h of treatment (Figure 4C). While mefloquine by itself did not cause an increase of lipidated LC3, there was an increase in the LC3-II lipidated isoform when mefloquine was combined with nelfinavir. It should be noted that the level of lipidated LC3 was not enhanced as much as chloroquine and nelfinavir treated *Tsc2*−/− MEFs, indicating that mefloquine is not as potent as chloroquine at inhibiting autophagy in these cells. Given the weaker level of autophagy inhibition with mefloquine, we considered it unlikely that blockade of autophagy was triggering the cell death response.

**Figure 5:**
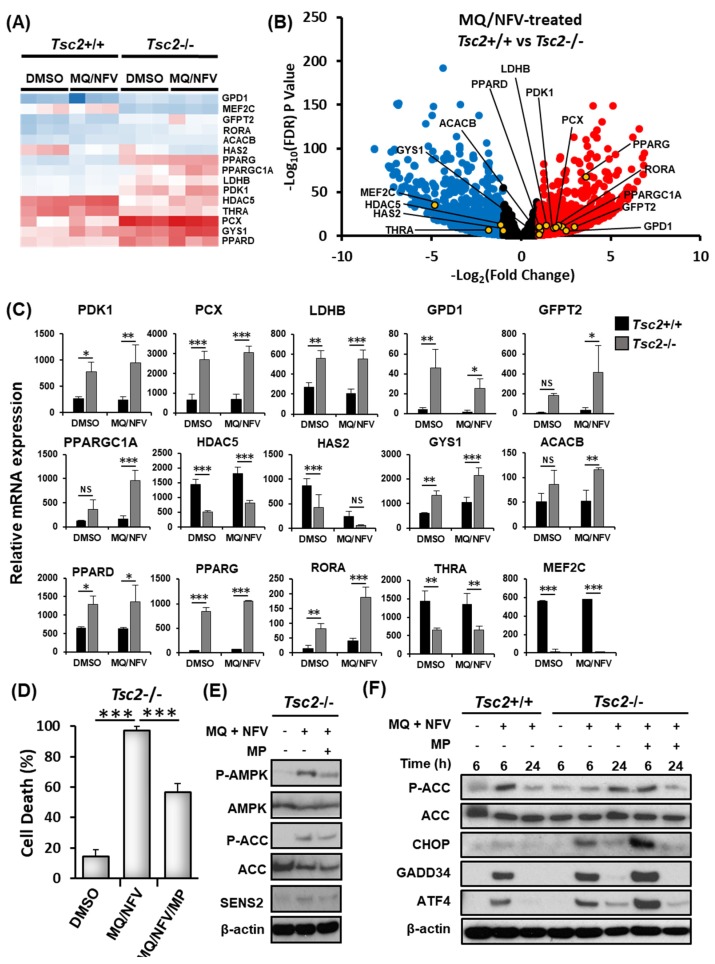
Mefloquine and nelfinavir combined drug treatment induces cytotoxicity via energy stress in *Tsc2*−/− MEFs. (**A**) The RNA sequencing data used for Figure 3C−E was assessed for gene-expression of genes involved in energy homeostasis. A heatmap for a panel of energy stress-linked genes is shown. Differences of mRNA expression between *Tsc2*+/+ and *Tsc2*−/− MEFs treated with mefloquine and nelfinavir is shown as a volcano plot (**B**) and graphed (**C**). (**D**) *Tsc2*−/− cells were treated with DMSO, 10 μM mefloquine and 10 μM nelfinavir combination (MQ/NFV) or mefloquine/nelfinavir combination with the addition of 8 mM methyl pyruvate (MQ/NFV/MP) for 48 h. Cells were then stained with DRAQ7 and % cell death determined by flow cytometry. (**E**) *Tsc2*−/− were treated with either DMSO or 10 μM mefloquine and 10 μM nelfinavir combination in the presence or absence of 8 mM methyl pyruvate for 24 h and total and phosphorylated ACC and AMPK was determined by western blot. (**F**) *Tsc2*+/+ and *Tsc2*−/− cells were treated with either DMSO or 10 μM mefloquine and 10 μM nelfinavir combination in the presence or absence of 8 mM methyl pyruvate for 6 and 24 h, where indicated. Total protein levels of ACC, CHOP GADD34 and ATF4 as well as phosphorylated ACC were detected by Western blot.

To explore alternative mechanisms that might cause cell death, the RNA sequencing data was further analyzed to compare gene expression changes in the *Tsc2*+/+ and *Tsc2*−/− MEFs (Figure 5A−C). Key genes involved in the regulation of metabolism and energy homeostasis were markedly up-regulated in the *Tsc2*−/− MEFs during combined treatment with nelfinavir and mefloquine. Of interest, PPARGC1α (typically referred to as PGC1α (peroxisome proliferator-activated receptor gamma coactivator 1α)) was basally expressed at a higher level in *Tsc2*−/− MEFs when compared to the *Tsc2*+/+ controls, which was further enhanced upon combined treatment with nelfinavir and mefloquine. Transcriptionally regulated genes of PGC1α were also observed to be more highly expressed in the *Tsc2*−/− MEFs when compared to wildtype. These included genes involved in glucose and lipid metabolism, such as peroxisome proliferator-activated receptor delta (PPARδ) and gamma (PPARγ) where there is more than a 2-fold increase in PPARδ gene expression in *Tsc2*−/− MEFs compared to *Tsc2*+/+ MEFS and a nearly 17-fold difference in PPARγ expression. Expression of genes involved in glycolysis were also upregulated, which is suggestive of metabolic stress. These genes included pyruvate dehydrogenase kinase 1 (PDK1), pyruvate carboxylase (PCX), lactate dehydrogenase B (LDHB) and glycerol-3-phosphate dehydrogenase 1 (GPD1). AMP-dependent protein kinase (AMPK) is known to function upstream of PGC1α, and is involved in the gene-expression of PGC1α as well as its activity [25]. AMPK-regulated genes involved in glucose metabolism/storage such as acetyl-CoA carboxylase 2 (ACC2, encoded by the ACACB gene) and glycogen synthase 1 (GYS1) were expressed at a much higher level in *Tsc2*−/− MEFs and were further increased upon drug treatment with nelfinavir and mefloquine. The overall increase of mRNA expression of key genes involved in energy metabolism indicates that the *Tsc2*−/− MEFs are likely energy stressed.

To determine whether energy stress could be a possible mechanism of cell death, flow cytometry with DRAQ7 labelling was carried out with samples treated for 48 h with either DMSO, or nelfinavir and mefloquine combination in the presence or absence of methyl pyruvate (Figure 5D). Methyl pyruvate can alleviate energy stress when supplemented to cells, as it is a direct substrate of the citric acid cycle to produce energy by oxidative phosphorylation [26]. Methyl pyruvate partially rescued nelfinavir and mefloquine induced cell death when supplemented to the *Tsc2*−/− MEFs (causing a 40% rescue of cell death). We confirmed that methyl pyruvate partially restored energy homeostasis within cells, as observed by a reduction in phosphorylated 5’-AMP-activated protein kinase (AMPK) and acetyl-CoA carboxylase (ACC). Methyl pyruvate also reduced the protein levels of SESN2 at the 24 h time point (Figure 5E). To further assess the capacity of *Tsc2*−/− MEFs to effectively restore energy stress, we examined phosphorylation levels of ACC after combined nelfinavir and mefloquine treatment at 6 h and 24 h time points (Figure 5F). In the presence of methyl pyruvate, the *Tsc2*−/− MEFs behaved similarly to the *Tsc2*+/+ MEFs after treatment with nelfinavir and mefloquine, where ACC phosphorylation was increased at 6 h and was then restored to a level equivalent to untreated cells at 24 h. In contrast, in the absence of methyl pyruvate the *Tsc2*−/− MEFs had a delayed induction of ACC phosphorylation during nelfinavir and mefloquine treatment. For instance, phosphorylation of ACC was only marginally enhanced at 6 h, but was then markedly elevated at 24 h. Expression of the ER stress proteins, ATF4, CHOP and GADD34 were significantly reduced at 24 h, showing recovery of ER stress. Collectively, our data shows that the *Tsc2*−/− MEF cells become energy stressed during the longer time points of nelfinavir and mefloquine treatment while they recover from ER stress.

## 3. Discussion

The central hypothesis of this study was to exploit the homeostatic vulnerabilities within TSC2-deficient cells with mTORC1 hyperactivity. The volume of unfolded protein within the ER is intensified by mTORC1 hyperactivity, caused by heightened levels of de novo protein translation and reduced efficiency of autophagy to remove the unfolded protein. Consequently, cell lines lacking TSC2 become more sensitive to drug treatments that induce ER stress. In this study, we employed nelfinavir as the ER stress inducer, and mefloquine as a potential autophagy inhibitor. We revealed that nelfinavir and mefloquine synergize to selectively kill TSC2-deficient cells. The concentrations of both drugs used in this study are clinically viable. For instance, 10 µM falls within the concentration range of mefloquine found in patient serum [27]. Regarding nelfinavir, the 10 µM concentration used in this study is higher than the manufacturer recommended trough concentration (1−3 µM). However, nelfinavir serum concentration has previously been reported in HIV patients at a similar concentration, ranging from 4.96 µM [28] up to 18 µM [29]. In fact, it has been reported that nelfinavir is well tolerated in cancer patients at doses 2.5 times the FDA-approved dose for HIV management [30]. We observed that the combination of nelfinavir and mefloquine had a long-lasting effect on *Tsc2*−/− tumor spheroids, when compared to single drug treatments. For instance, with mefloquine, despite an increase in DRAQ7 staining, indicating a level of cell death, cell recovery and outgrowth from the bulk tumor after drug withdrawal was apparent. However, no cells recovered when nelfinavir was combined with mefloquine. As well as cells lacking *Tsc2*, the nelfinavir and mefloquine drug combination was effective at killing sporadic cancer cell lines with aberrant signaling through the TSC1/TSC2 signaling axis, with MCF7 cells being much more sensitive to the dual therapy when compared to single drug treatments.

Through transcriptional profiling of nelfinavir and mefloquine treated cells, we observed an ER stress signature at the early 6 h time-point of treatment, implying that ER stress might be involved in the observed cytotoxic response. However, when we examined the longer time points of combined nelfinavir and mefloquine treatment, we observed recovery of the ER stress ATF4-CHOP signaling pathway within the *Tsc2*−/− MEF cells. ER stress-mediated cell death occurs when the protein level of CHOP remains persistently elevated over a long time course of drug treatment. Given the temporary elevation of CHOP in the continued presence of drug in both the *Tsc2*+/+ and *Tsc2*−/− MEFs, our data indicates a good level of ER stress recovery and excludes the possibility that CHOP triggers cell death.

Further analysis of the RNA sequencing data in the *Tsc2*−/− MEFs implied that energy homeostasis pathways were elevated after co-treatment with nelfinavir and mefloquine. We rescued a significant degree of cell death with methyl pyruvate, indicating that cytotoxicity by nelfinavir and mefloquine was at least partially mediated through energy deficiency. Methyl pyruvate is a methyl ester of pyruvic acid that restores cellular energy production downstream of glycolysis by acting as a mitochondrial citric acid cycle substrate. While ER stress is unlikely the trigger of cell death, recovery from ER stress might contribute to the depletion of energy through the de novo protein synthesis of chaperone and heat shock proteins that are required for the unfolding and refolding of protein aggregates in the ER, processes that heavily consume ATP. A recent study showed that TSC2-knockdown leads to mitochondrial oxidative stress [31]. This degree of energy stress is presumably why TSC2-deficent cells are vulnerable to conditions that induce energy starvation [32].

Mefloquine has shown promise as an anti-cancer drug in several cancer model settings. Liu et al. showed that mefloquine (whether as a single agent or in combination with paclitaxel) induced apoptosis in gastric cancer cell lines and gastric cancer xenograft mouse models [23]. PC3 cells, a prostate cancer cell line, were also sensitive to mefloquine at 10 µM after 24 h of treatment [22]. However, no further toxicity was detected between 24 h to 72 h of mefloquine treatment, suggesting that mefloquine may not be fully effective as a monotherapy. Mefloquine was shown to inhibit autophagy, trigger ER stress and induced cell death in human breast cancer lines, and was more cytotoxic when compared to chloroquine [33]. Our data is in line with these previous studies, except we observed weaker inhibition of autophagy with mefloquine treatment. We further enhanced the cytotoxic effectiveness of mefloquine with dual treatment with nelfinavir and determined that cell death was caused by an imbalance in energy homeostasis. In the cancer setting, mefloquine is currently in a phase I clinical factorial trial (NCT01430351) in combination with the DNA-damaging agent temozolomide for glioblastoma multiforme. It should be noted that mefloquine has advantages over chloroquine for the treatment of brain cancers, as mefloquine has better penetration through the blood brain barrier. In summary, we show that mefloquine synergizes with nelfinavir to induce a high degree of cell death through energy stress. There is clinical application for this drug combination in the cancer setting, where we can exploit the lack of flexibility that cancer cells have in restoring homeostatic balance.

## 4. Materials and Methods 

### 4.1. Cell Culture and Drug Treatments

Tsc2-null ELT3-V3 and control ELT3-T3 cells re-expressing TSC2 were kindly provided by Cheryl Walker (M.D. Anderson Cancer Centre, Houston, USA). *Tsc2*+/+ *p53*−/− and *Tsc2*−/− *p53*−/− MEFs were kindly provided by David J. Kwiatkowski (Harvard University, Boston, MA, USA). Human breast cancer (MCF7), human colorectal cancer (HCT116) and human lung carcinoma (NCI-H460) cell lines were purchased from ATCC. All cell lines were routinely tested using the Venor GeM Classic PCR kit from (CamBio) and were clear of mycoplasma. Cells were cultured in Dulbecco’s Modified Eagle’s Medium (DMEM), while the MCF7 cell line was incubated in Roswell Park Memorial Institute (RPMI) 1640. Cell media was supplemented with 10% (v/v) Fetal Bovine Serum (FBS) and 100 U/mL Penicillin and 100 μg/mL Streptomycin (Life Technologies Ltd., Paisley, UK). All cell lines were incubated at 37 °C, 5% (v/v) CO_2_ in a humidified incubator. Nelfinavir mesylate hydrate, chloroquine di-phosphate salt, mefloquine hydrochloride, rapamycin, etoposide and thapsigargin were purchased from Sigma Aldrich Ltd. (Gillingham, Dorset, UK). DMSO was used as a solvent to make stock solutions of etoposide (100 mM), mefloquine (50 mM), nelfinavir (30 mM), thapsigargin (10 mM) and rapamycin (100 µM). Chloroquine was made fresh in culture medium at a 100 mM stock and further diluted in culture medium to the required concentration before use. Drug(s) dissolved in DMSO or DMSO vehicle control were added to cells in culture media so that they were diluted to a final concentration that was below 0.8% (v/v) DMSO.

### 4.2. Flow Cytometry

Treated cells were collected and incubated with 3 µM DRAQ7 (Biostatus, Leicestershire, UK) at room temperature for 10 min. Flow cytometry was performed using a FACS Calibur flow cytometer (Becton Dickinson (Cowley, UK)) with excitation set at 488 nm and detection of fluorescence in log mode at wavelengths greater than 695 nm. Cell Quest Pro software was used for signal acquisition. A total of 10,000 events per sample were collected.

### 4.3. Western Blotting

S6K1, phospho-S6K1(Thr389), rpS6, phospho-rpS6(Ser235/236), IRE1α, CHOP, GADD34, ATF4, TSC2, SESN2, AMPK, phospho-AMPK(Thr172), ACC, phospho-ACC (Ser79) and β-actin antibodies were purchased from Cell Signaling Technology (Danvers, USA). LC3 antibodies were bought from Novus Ltd. (Cambridge, UK). Cells were washed in ice-cold phosphate buffered saline (PBS) and then lysed in cell lysis buffer (20 mM Tris (pH 7.5), 125 mM NaCl, 50 mM NaF, 5% (v/v) glycerol, 0.1% (v/v) Triton X-100, supplemented with 1 mM dithiothreitol (DTT), 1 µg/mL pepstatin, 20 µM leupeptin, 1 mM benzamidine, 2 µM antipain, 0.1 mM PMSF, 1 mM sodium orthovanadate and 1 nM okadaic acid prior to cell lysis). Cell lysates were sonicated using a diagenode bioruptor (Diagenode, Seraing, Belgium) and centrifuged at 13,000 rpm for 8 min. Protein concentrations were determined by a Bradford assay (Thermo Fisher Scientific, Paisley, UK). Samples were diluted in 4 × NuPAGE loading sample buffer (Life Technologies) with 25 mM DTT and boiled at 70 °C for 10 min. Western blot was performed as previously described [34].

### 4.4. mRNA Extraction and Reverse Transcription

Treated cells were washed in ice cold PBS then lysed using RNAprotect Cell Reagent (Qiagen, West Sussex, UK). RNA concentrations were determined by measuring the absorbance at 260 nm and 280 nm in a Nanodrop spectrophotometer. Total RNA from each sample (1 μg) was converted to copy DNA (cDNA) using the Quantitect reverse transcription kit (Qiagen) following the manufacturer’s protocol.

### 4.5. XBP-1 Splicing

Protocol for XBP-1 splicing is as described in reference [19]. *Xbp1* primers (forward: 5′-AAA CAG AGT AGC AGC TCA GAC TGC-3′, reverse: 5′-TCC TTC TGG GTA GAC CTC TGG GA-3′, were synthesized by MWG Operon-Eurofin (Ebersberg, Germany)). β-actin primers were purchased from Qiagen (QT01136772). PCR was performed in an Applied Biosystems GeneAmp 9700 PCR system in the following conditions: Initial denaturation step (94 °C, 3 min); 31 cycles of denaturation (94 °C, 45 s); annealing step (60 °C, 30 s); extension step (72 °C, 1 min); final extension step (72 °C, 10 min). A 3% (w/v) agarose (Appleton, Birmingham, UK) 1 × Tris-acetate-EDTA (4.84 g Tris-base (pH 8.0), 0.372 g EDTA and 1.7 mL acetic acid in 1 L deionized water) was made with 0.005% (v/v) GelRed nucleic acid stain (Biotium, Fremont, CA, USA). DNA samples were loaded with Orange G loading buffer (15 mL 30% (v/v) glycerol, 100 mg Orange G powder, deionized water, total volume 50 mL) and resolved on the gel at 100 V. After 1 h, β-actin samples were analyzed. Samples were resolved for an additional 2 h for XBP-1 splicing. PCR products of *Xbp1* were 480 bp (unspliced) and 454 bp (spliced). 

### 4.6. Tumor Formation Assay

A 1.2% (w/v) agar solution in PBS (Difco Agar Noble (BD, Oxford, UK)) was diluted with DMEM to a final concentration of 0.6% (w/v) then added to a 6 well plate and allowed to set. A total of 150,000 *Tsc2*−/− MEF cells were added to a 0.3% (w/v) agar-PBS:DMEM mixture and 3 mL was layered on top and left to set. Plates were incubated overnight with 2 mL of DMEM. The following day, the 2 mL of DMEM was replaced with DMEM containing either drugs or DMSO. Media and drugs were refreshed every 48–72 h for 14 days. Images were taken using an EVOS XL Core camera (Life Technologies). Tumor diameter was analyzed using Image J.

### 4.7. Tumor Outgrowth Assay

A total of 70 µl of 1.5% (w/v) agarose-PBS was added to each well of a 96 well plate and allowed to set. A total of 1000 *Tsc2*−/− MEF cells in 140 μL complete DMEM were added to each well. Spheroids were formed over 72 h. Following formation, spheroids were drug treated for 48 h. Cells were imaged and then treated for 48 h. Treatment was refreshed and a final concentration of 3 µM DRAQ7 was added to each well and incubated for an additional 48 h. Imaging and subsequent outgrowth was performed as previously described [35].

### 4.8. RNA Sequencing

Total RNA quality and quantity was assessed using Agilent 2100 Bioanalyser and an RNA Nano 6000 kit (Agilent Technologies, Cheshire, UK). A total of 100–900 ng of total RNA with a RIN value >8 was used as the input and the sequencing libraries were prepared using the Illumina^®^ TruSeq^®^ RNA sample preparation v2. (Illumina Inc., Cambridge, UK). The steps included two rounds of purification of the polyA containing mRNA molecules using oligo-dT attached magnetic beads followed by RNA fragmentation, 1st strand cDNA synthesis, 2nd strand cDNA synthesis, adenylation of 3’-ends, adapter ligation, PCR amplification (15 cycles) and validation. The manufacturer’s instructions were followed. The libraries were validated using the Agilent 2100 Bioanalyser and a high-sensitivity kit (Agilent Technologies) to ascertain the insert size, and the Qubit^®^ (Life Technologies) was used to perform the fluorometric quantitation. Following validation, the libraries were normalized to 4 nM and pooled together. The pool was then sequenced using a 75 base paired-end (2 × 75 bp PE) dual index read format on the Illumina^®^ HiSeq2500 in rapid mode according to the manufacturer’s instructions. The pool was then sequenced using a 75 base paired-end (2 × 75 bp PE) dual index read format on the Illumina^®^ HiSeq2500 in high-output mode according to the manufacturer’s instructions. Quality control checks of the resultant reads were performed using FastQC before mapping to the UCSC mouse mm10 reference genome using Tophat and Bowtie. Differentially expressed transcripts were identified using a DeSeq2 analysis [36] on normalized count data with the design formula setup to analyze all pairwise comparisons in the dataset using contrasts. The resultant *p*-values were corrected for multiple testing and false discovery issues using the FDR method. Genes involved in cell survival were selected based on GO: 0008219 (cell death) from the complete list on AmiGo 2.

### 4.9. Statistical Analysis

All experiments were carried out with a minimum of three biological repeats. Where applicable, results are written as mean with a +/− St-Dev. Either a two-way ANOVA (with Bonferroni’s multiple comparison post-hoc test) or one-way ANOVA (with Tukey’s multiple comparison post-hoc test) was used to determine statistical significance. Significance was reported as a *p* value: * *p* < 0.05, ** *p* < 0.01, *** *p* < 0.001.

## 5. Conclusions

In conclusion, mefloquine synergizes with nelfinavir to induce a high degree of cell death through energy stress. There is potential to use mefloquine with nelfinavir in the cancer setting, to treat cancer cells that have a lack of flexibility in restoring homeostatic balance.

## Supplementary Materials

The following are available online at http://www.mdpi.com/2072-6694/10/10/375/s1, Table S1: RNAseq raw data of mefloquine and nelfinavir treatment of *Tsc2* MEFs, Table S2: RNAseq of *Tsc2*−/− compared to *Tsc2*+/+ after mefloquine and nelfinavir treatment.
